# Changes in Susceptibility Profiles of *Acinetobacter baumannii* Clinical Isolates in a Multi-Profile Hospital in Years 2020–2024 in Lodz, Poland

**DOI:** 10.3390/jcm15093505

**Published:** 2026-05-03

**Authors:** Adrian Bekier, Filip Bielec, Magdalena Grędysa, Eliza Miaśkiewicz, Małgorzata Nowak, Dorota Pastuszak-Lewandoska, Małgorzata Brauncajs

**Affiliations:** 1 Department of Microbiology and Medical Laboratory Immunology, Medical University of Lodz, 92-213 Lodz, Poland; adrian.bekier@umed.lodz.pl (A.B.); filip.bielec@umed.lodz.pl (F.B.); malgorzata.nowak3@student.umed.lodz.pl (M.N.); dorota.pastuszak-lewandoska@umed.lodz.pl (D.P.-L.); 2 Laboratory of Medical Microbiology, Central Teaching Hospital of Medical University of Lodz, 92-213 Lodz, Poland; mgredysa@csk.umed.pl (M.G.); emiaskiewicz@csk.umed.pl (E.M.); 3 Department of Epidemiology, Central Teaching Hospital of Medical University of Lodz, 92-213 Lodz, Poland

**Keywords:** *Acinetobacter baumannii*, antimicrobial resistance, carbapenem resistance, MDR, CRAB, Poland, susceptibility patterns

## Abstract

**Background**: *Acinetobacter baumannii* is a non-fermenting Gram-negative bacillus responsible for severe nosocomial infections, particularly in intensive care units (ICUs). The increasing prevalence of multidrug-resistant (MDR) and carbapenem-resistant *A. baumannii* (CRAB) strains has become a significant challenge for infection control and antimicrobial therapy worldwide. **Objectives**: This study aimed to analyze the antimicrobial susceptibility patterns of clinical *A. baumannii* isolates recovered from a multi-profile hospital in years 2020–2024 in Lodz, Poland. **Methods**: Clinical isolates from various specimen types (blood, urine, wound swabs, biopsies, sputum, and bronchoalveolar lavage fluid) were obtained during routine microbiological diagnostics. Identification was performed using MALDI-TOF MS. Antimicrobial susceptibility testing (AST) was conducted using the automated VITEK^®^2 system with EUCAST/CLSI interpretive criteria. Minimum inhibitory concentrations (MICs) for colistin were determined by broth microdilution. Carbapenemase production was assessed using the Carbapenem Inactivation Method (CIM) and immunochromatographic assays for OXA-23, OXA-40/58, and NDM detection. **Results**: A total of 244 *A. baumannii* isolates were recovered over the study period. Susceptibility to carbapenems (meropenem, imipenem) declined markedly, with resistance exceeding 90% by 2023–2024. Aminoglycosides exhibited variable activity, with gentamicin demonstrating the highest susceptibility rates (up to 88% in 2022). Resistance to ceftazidime and cefepime remained consistently high (>90% in 2023–2024). No fully susceptible isolates were identified for ciprofloxacin. **Conclusions**: The high prevalence of CRAB strains highlights the urgent need for effective infection control measures, optimized antimicrobial stewardship, and consideration of novel treatment options in the clinical setting.

## 1. Introduction

### 1.1. Epidemiology

The *Acinetobacter baumannii* (*A. baumannii*) is a Gram-negative, non-fermenting bacillus responsible for healthcare-associated infections (HAIs), particularly in intensive care units (ICUs). Considering the recent rise in infections caused by this organism, it is critical to understand multidrug-resistant strains of *A. baumannii*, especially carbapenem-resistant isolates (CRAB; carbapenem-resistant *Acinetobacter baumannii*), to enable effective infection-control strategies and therapy for critically ill patients [1,2,3].

Infections due to *Acinetobacter* spp. display diverse epidemiology. They include nosocomial outbreaks in temperate climates as well as infections associated with natural disasters, armed conflicts, and tropical environments. *A. baumannii* is found predominantly in healthcare settings, very frequently in neonatal and adult ICUs, and on surgical, neurosurgical, internal medicine, oncology, and burn units. The organism is most often isolated from the respiratory tract, blood, and the central nervous system. It is also implicated in catheter-associated urinary tract infections and surgical site infections [3,4]. A common feature across these scenarios is disruption or damage to anatomic barriers, which enables bacteria to enter the host directly. *A. baumannii* causes a wide spectrum of HAIs, with the most frequent being bacteremia and ventilator-associated pneumonia (VAP) [4].

Due to its ability to form biofilms on synthetic materials, particularly endotracheal tubes, *A. baumannii* can extensively colonize the lower respiratory tract, leading to ventilator-associated pneumonia. Reports from the CDC indicate an increase in hospital-acquired infections caused by carbapenem-resistant *A. baumannii*, particularly during periods of healthcare system strain, such as the COVID-19 pandemic [5].

According to the European Centre for Disease Prevention and Control (ECDC), in 2023, 28 EU/EEA countries reported 8973 invasive *Acinetobacter* spp. isolates [6]. Four member states reported fewer than 30 isolates; Liechtenstein and France were not included because they submitted no data to the EpiPulse system. Among countries that reported continuously from 2019 to 2023 (excluding France due to changes in national surveillance), the number of reported invasive *Acinetobacter* spp. isolates increased by 84.6%. However, compared with the peak number reported between 2019 and 2023 (in 2021; *n* = 10,707), the 2023 count was lower. Moreover, although numbers rose between 2022 and 2023, the increase was only 1.7%. The estimated incidence of invasive *Acinetobacter* spp. infections in EU/EEA countries rose by 21.1%; nevertheless, compared with the 2021 peak, the incidence in 2023 decreased by 24.6% [6].

As of 2018, *A. baumannii* accounted for ~2% of all HAIs in the USA and EU/EEA, whereas in Asia and the Middle East the proportion was nearly double [7]. Although *A. baumannii* may cause infections at rates comparable to other Gram-negative bacteria, about 45% of isolates are multidrug-resistant four times the rate observed for Klebsiella pneumoniae or Pseudomonas aeruginosa. In Latin America and the Middle East, these proportions can reach 70%. Nosocomial *A. baumannii* infections are associated with an 8–40% increase in mortality risk [2,4].

*A. baumannii* can multiply on nutrient-poor surfaces and is introduced into the hospital environment by colonized patients. It can colonize many elements in the patient’s surroundings, including linens, furniture, sinks, and medical equipment. While droplet transmission or contact with colonized skin is possible, the principal route of spread is via the hands of healthcare workers. Patients may carry the organism for many days or even weeks before *A. baumannii* is detected by screening [3,6].

### 1.2. Virulence Factors

*A. baumannii* possesses multiple virulence factors, including outer membrane proteins, capsule formation, and secretion systems, which contribute to host colonization, immune evasion, and persistence in hospital environments. These features facilitate its survival and transmission in healthcare settings [8,9,10,11].

### 1.3. Resistance Mechanisms

Resistance in *A. baumannii* is mediated by multiple mechanisms, including the production of β-lactamases, reduced outer membrane permeability, and efflux pump overexpression [12]. Among these, class D oxacillinases (OXA-type enzymes) represent the predominant mechanism of carbapenem resistance, while metallo-β-lactamases (class B) are reported less frequently [13]. The most frequently identified types are OXA-23, OXA-24/40, OXA-51, OXA-58, OXA-143, and OXA-235, with worldwide clinical detection. In the USA and EU/EEA, OXA-23, OXA-24/40, and OXA-58 predominate. OXA-23 is the most common cause of carbapenem resistance [14]. The coexistence of several resistance mechanisms contributes to the emergence of multidrug-resistant and extensively drug-resistant phenotypes [3].

### 1.4. Carbapenem-Resistant Acinetobacter baumannii (CRAB)

In WHO’s 2024 list of priority bacterial pathogens, Gram-negative bacteria remain in the highest-priority category [15]. The “critical priority” group includes carbapenem-resistant *A. baumannii* (CRAB), carbapenem-resistant *Enterobacterales* (CRE), and third-generation cephalosporin-resistant *Enterobacterales* (3GCRE) [15,16].

CRAB poses a major global challenge due to high virulence, multidrug resistance, and limited therapeutic options. It causes severe nosocomial infections, especially among ICU patients, and is associated with very high mortality [16,17]. CRAB ranks among the five pathogens with the greatest number of deaths attributable to antimicrobial resistance worldwide and is the leading cause of mortality in Southeast Asia, East Asia, and Oceania [16,18,19].

Despite urgent need, antibiotic development against CRAB has been inadequate. Since CRAB was designated a critical-priority pathogen on the 2017 WHO list, no new agents effective against MBL-producing strains have been introduced, underscoring the difficulty of the problem and the need for further research [20,21].

Epidemiological studies show worrisome increases in CRAB infections; global mortality ranges from 27.8% to 35% [15]. In low- and middle-income countries, the burden is driven by such factors as lack of systematic surveillance, insufficient implementation of infection-prevention and control protocols, misuse and overuse of antibiotics, and under-resourced healthcare infrastructure [22]. Data gaps especially among ICU patients in sub-Saharan Africa represent a major knowledge deficit that must be addressed to design effective, evidence-based interventions and health policies [23].

Unregulated over-the-counter antibiotic sales, widespread antimicrobial use in hospitals, poor hand hygiene, and absent basic infection-control measures (isolation, personal protective equipment) exacerbate antimicrobial resistance in developing countries. Introduction of a single CRAB clone into a healthcare facility, followed by spread due to infection-control breaches, often leads to hospital outbreaks even in high-income settings. Such clonal outbreaks can usually be contained through epidemiological investigations and targeted countermeasures. Genotyping via next-generation sequencing or biotyping enables precise characterization of intra-hospital epidemics [24,25]. The current pandemic of resistance among Gram-negative bacteria can be mitigated through rigorous infection-control policies and antimicrobial stewardship.

In hospitals, CRAB can spread rapidly and be difficult to eradicate; therefore prompt implementation of preventive procedures is essential. Effective control requires close collaboration among the Microbiology Laboratory, clinical departments, and the Infection Prevention and Control team [26]. Hospitals should utilize data from national public health authorities (e.g., State Sanitary Inspection) and the National Reference Centre for Antimicrobial Susceptibility (KORLD, Poland), including epidemiological characteristics and resistance mechanisms.

### 1.5. Treatment

#### 1.5.1. First-Line Antibiotics

Among classical β-lactams, ceftazidime, cefepime, piperacillin-tazobactam, and ampicillin-sulbactam may retain activity against susceptible *Acinetobacter* spp.; however, their utility is limited due to the high prevalence of multidrug resistance. Ampicillin-sulbactam is notable because sulbactam, the β-lactamase inhibitor, exhibits intrinsic bactericidal activity against this pathogen. A meta-analysis of 18 studies including 1800 patients found that combination regimens containing high-dose ampicillin–sulbactam (≥6 g/day) did not demonstrate a significant reduction in mortality compared with colistin-based regimens; however, they were associated with a lower risk of nephrotoxicity [27]. Another meta-analysis (23 studies) likewise found lower mortality with sulbactam-containing regimens versus polymyxin- or tigecycline-based therapies [28]. Infectious Diseases Society of America (IDSA) recommends high-dose ampicillin-sulbactam as the backbone of therapy for severe CRAB infections, as monotherapy or in combination, used only when sulbactam–durlobactam is unavailable [2].

High-dose carbapenems (imipenem, meropenem) administered as prolonged infusions are used for serious infections caused by multidrug-resistant Gram-negative bacteria, typically in combination with other agents, while ertapenem lacks activity against *A. baumannii*. Time-dependent pharmacodynamics favor extended infusions [2].

Fluoroquinolones (ciprofloxacin, levofloxacin), administered orally or parenterally, can be effective against susceptible *A. baumannii* strains, as monotherapy or in combination depending on infection severity [2].

Aminoglycosides (amikacin, gentamicin) are most often used for urinary tract infections (UTIs) without bacteremia because of the very high urinary concentrations achieved [29]. Trimethoprim–sulfamethoxazole may be considered for the treatment of UTIs if susceptibility is confirmed, though most isolates are resistant [30].

#### 1.5.2. Second-Line Antibiotics

Sulbactam–durlobactam combines two β-lactamase inhibitors, sulbactam and durlobactam, a novel diazabicyclooctane derivative. This agent is the preferred treatment for CRAB and is recommended in combination with imipenem or meropenem [2]. It received U.S. FDA approval in May 2023 but is not available in Poland. Durlobactam strongly inhibits class A, C, and D β-lactamases but not class B MBLs, protecting sulbactam from hydrolysis and allowing it to reach its PBP targets [31]. The dose is 1 g sulbactam plus 1 g durlobactam (total 2 g) every 6 h as a 3-h IV infusion [32]. This regimen achieves PK/PD targets for >90% of *A. baumannii* isolates with sulbactam–durlobactam MIC ≤ 4/4 µg/mL, consistent with CLSI breakpoints [32]. Efficacy was evaluated in a clinical trial in patients with pneumonia or bacteremia caused by *A. baumannii* [33].

Polymyxins are used when first-line agents cannot be used. Polymyxin E (colistin) is administered clinically as the prodrug colistimethate sodium, which is hydrolyzed to active colistin after parenteral administration. Polymyxin E is preferred for UTIs, whereas polymyxin B is used for other sites; both have limited pulmonary penetration. Despite limited lung penetration, colistin has performed similarly to other agents for VAP treatment [34]. Many studies have used both parenteral and inhaled (nebulized) colistin. The principal adverse effect is nephrotoxicity. To ensure efficacy against susceptible strains, plasma colistin concentrations should be ≥2 mg/L [35].

Cefiderocol, a siderophore cephalosporin, is an option for highly resistant CRAB strains. It has not demonstrated superiority over other therapies [36], but many CRAB isolates, including those producing class D β-lactamases, remain susceptible [37,38].

Tetracyclines are considered second-line agents. Minocycline (oral and IV) has shown clinical and microbiological effectiveness in retrospective studies [39]; it can be used as monotherapy for mild infections or in combination for severe disease. Doxycycline is another option but with lower susceptibility rates. Eravacycline is used mainly for intra-abdominal infections; it may be considered when first- and second-line options fail. Although it is active in vitro against CRAB, the absence of established breakpoints complicates interpretation, and the drug is not formally approved for these infections [40].

Tigecycline, a glycylcycline and semisynthetic tetracycline analog available only IV, has activity against *Acinetobacter* spp. Higher dosing (100 mg IV q12h) yields better clinical outcomes than conventional dosing (50 mg IV q12h). Tigecycline is an option for infections not involving the bloodstream or urinary tract [41].

#### 1.5.3. Combination Therapy

Combination therapy is recommended for moderate to severe infections. In settings with high local resistance, using multiple agents increases the chance that at least one will be active and may delay further resistance development. However, clinical trials have not consistently demonstrated its superiority [42]. Evaluated combinations for multidrug-resistant CRAB include:

- Colistimethate sodium plus rifampicin vs. colistimethate monotherapy: no mortality benefit in a study of 210 patients [43].
- Colistimethate sodium plus rifampicin vs. monotherapy: no significant mortality reduction in 43 patients with VAP [42].
- Colistimethate sodium plus fosfomycin vs. monotherapy: no mortality benefit in 94 patients with various CRAB infections [44].
- Colistimethate sodium plus meropenem vs. monotherapy: no mortality reduction in a large trial of 312 patients [45].
- Colistimethate sodium plus ampicillin–sulbactam vs. monotherapy: earlier clinical improvement with combination therapy in a study of 39 patients with CRAB [46].
- Sulbactam–durlobactam vs. colistimethate monotherapy: comparable 28-day mortality in 125 patients with CRAB infections [33].

Despite the lack of definitive evidence favoring combination therapy over monotherapy, it may be justified in critically ill patients due to the risks of host immune failure, rapid selection of resistant subpopulations, and heteroresistance that routine microbiology may miss [2].

The aim of this study was to analyze temporal trends in antimicrobial susceptibility patterns of *Acinetobacter baumannii* clinical isolates collected from a multi-profile hospital in Łódź, Poland, between 2020 and 2024.

## 2. Materials and Methods

### 2.1. Bacterial Strains

The analysis included *A. baumannii* clinical isolates recovered from various specimens collected between January 2020 and December 2024. All isolates originated from routine microbiological diagnostics performed at the Medical Microbiology Laboratory.

### 2.2. Microbiological Assays

#### 2.2.1. Identification of Isolates

Bacterial identification was performed using the VITEK^®^ MS system (bioMérieux, Marcy-l’Étoile, France), based on matrix-assisted laser desorption/ionization time-of-flight mass spectrometry (MALDI-TOF MS) technology. In cases where the identification confidence was below the manufacturer’s threshold, species confirmation was carried out using the VITEK^®^ 2 automated system with GN identification cards (bioMérieux, Marcy-l’Étoile, France), according to the manufacturer’s recommendations.

#### 2.2.2. Antimicrobial Susceptibility Testing (AST)

AST of *A. baumannii* isolates was performed using the VITEK^®^ 2 automated system (bioMérieux, Marcy-l’Étoile, France) with the AST-N331 card designed for non-fermenting Gram-negative bacilli. The following antimicrobial agents were tested: ceftazidime, cefepime, imipenem, meropenem, gentamicin, amikacin, tobramycin, ciprofloxacin, levofloxacin, trimethoprim–sulfamethoxazole, and tigecycline. The isolates used for AST were derived from pure overnight cultures grown on Columbia agar with 5% sheep blood at 35 °C for 18–20 h. Inocula were prepared from at least five colonies suspended in 0.85% saline to achieve a turbidity equivalent to 0.50–0.63 McFarland. The results were automatically interpreted by the VITEK^®^ 2 system and categorized according to the European Committee on Antimicrobial Susceptibility Testing (EUCAST) clinical breakpoints valid for each study year (versions 10.0–14.0) or Clinical and Laboratory Standards Institute (CLSI) M100 Performance Standards for Antimicrobial Susceptibility Testing (30th–34th editions).

#### 2.2.3. Determination of Colistin MIC

Determination of the minimum inhibitory concentration (MIC) for colistin was performed for carbapenem-resistant *A. baumannii* isolates using the ComASP™ Colistin 0.25–16 µg/mL test (Liofilchem^®^, Roseto degli Abruzzi, Italy), following the manufacturer’s protocol. Bacterial suspensions were prepared from pure overnight cultures grown on Columbia agar with 5% sheep blood at 35 °C. The test was incubated at 35 °C for 16–20 h, and MIC values were visually determined as the lowest concentration of colistin inhibiting visible bacterial growth. The obtained MIC results were interpreted according to the EUCAST clinical breakpoints valid for each study year.

#### 2.2.4. Detection of Carbapenemase Production

Carbapenemase production among carbapenem-resistant *A. baumannii* isolates was assessed using two complementary methods. First, the Carbapenem Inactivation Method (CIM) was performed according to the recommendations of the Polish National Reference Centre for Susceptibility Testing (KORLD). Briefly, bacterial suspensions prepared in distilled water from pure overnight cultures were incubated with a meropenem (10 µg) disk at 35 °C for 4 h. The disk was then placed on a Mueller–Hinton agar plate inoculated with the indicator strain Escherichia coli ATCC 25922. After incubation at 35 °C for 18–24 h, the inhibition zone around the disk was measured. A reduction in the inhibition zone (≤17 mm) was interpreted as a positive result, indicating carbapenemase activity. To identify the specific type of carbapenemase, the RESIST-ACINETO immunochromatographic test (Coris BioConcept^®^, Gembloux, Belgium) was used, allowing detection of OXA-23, OXA-40/OXA-58, and NDM enzymes. The test was performed and interpreted according to the manufacturer’s instructions.

### 2.3. Statistical Analysis

All statistical analyses were performed using GraphPad Prism version 10.0 (GraphPad Software, San Diego, CA, USA). Annual antimicrobial resistance rates were analyzed separately for each antibiotic and antibiotic class.

For all agents, including cephalosporins, carbapenems, aminoglycosides, fluoroquinolones, trimethoprim–sulfamethoxazole, and tigecycline, linear regression analysis was used to evaluate trends in resistance between 2020 and 2024, with the percentage of resistant isolates as the dependent variable and year as the independent variable. In cases of non-normal data distribution or irregular patterns, the Spearman rank correlation test was applied to assess the direction and significance of monotonic changes.

For colistin, resistance was defined according to EUCAST clinical breakpoints (MIC ≤ 2 mg/L = susceptible; >2 mg/L = resistant). The proportion of resistant CRAB isolates was calculated for each study year, together with 95% confidence intervals (CIs) using the Clopper-Pearson exact method. The presence of a linear trend across the five-year period was evaluated using the Cochran-Armitage trend test, while logistic regression was additionally applied to estimate the odds ratio (OR) for resistance per year with 95% CI.

### 2.4. Ethical Issues

This study follows the 1964 Helsinki declaration and its later amendments. The consent of the Bioethics Committee was not required for this study. The study involved only anonymized records and identification of a specific person was not possible. All bacterial strains were previously secured in the culture collection of our research unit, with the use of consecutive code identification numbers. We only possess clinical data on the sex and age of the patients and the type of biological material from which the bacterial strain was isolated.

## 3. Results

### 3.1. Characteristics of Acinetobacter baumannii Clinical Isolates

**Figure 1:**
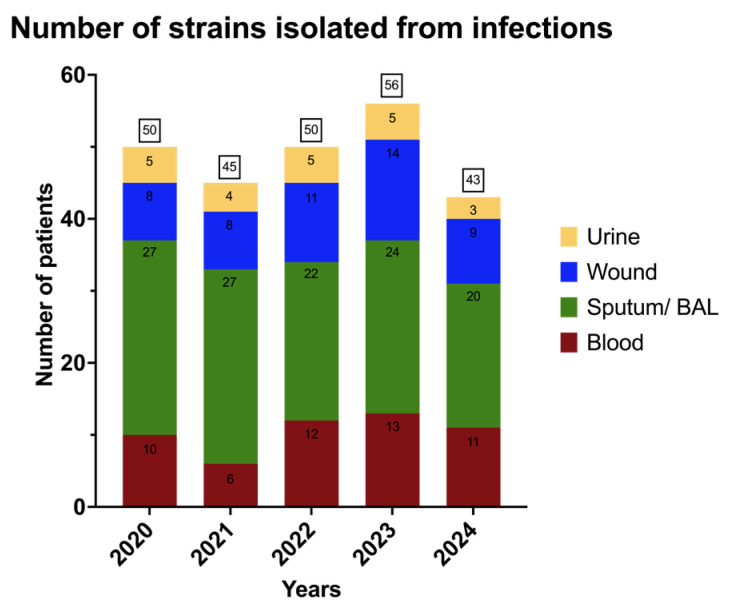
Annual distribution of *Acinetobacter baumannii* isolates from various clinical specimens (e.g., urine, blood, wound swabs, biopsies, sputum, and bronchoalveolar lavage) in 2020–2024.

A total of 244 non-duplicate *A. baumannii* strains were included in the analysis. The majority of isolates were obtained from respiratory tract specimens (120/244; 49.2%), followed by blood (52/244; 21.3%), wound-related samples (50/244; 20.5%), and urine (22/244; 9.0%). Only the first isolate per patient per infection episode was included in the analysis to avoid duplication. The annual distribution of isolates, stratified by specimen type, is presented in Figure 1.

The study population comprised 244 patients, with a clear predominance of males (73%; M/F ≈ 2.7). The cohort consisted exclusively of adult patients, with ages ranging from 21 to 57 years (median 47 years). Isolates were recovered from patients hospitalized in diverse clinical settings, including internal medicine, intensive care, cardiology, nephrology, and surgical wards. Among them, 201 (82.4%) were classified as multidrug-resistant (MDR), 32 (13.1%) as extensively drug-resistant (XDR), and 11 (4.5%) as pandrug-resistant (PDR).

### 3.2. β-Lactams

**Figure 2:**
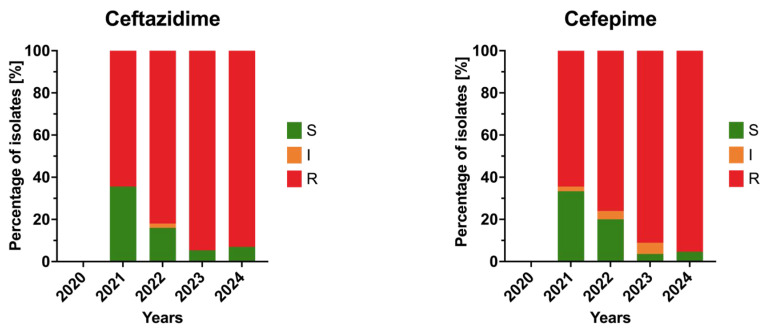
Annual resistance rates of *Acinetobacter baumannii* clinical isolates to cephalosporins in 2020–2024. Bars represent proportions of susceptible (S), susceptible increased exposure (I), and resistant (R) isolates.

Among cephalosporins, both ceftazidime and cefepime were introduced into routine testing from 2021 onward. A sharp increase in resistance was observed for both agents over the subsequent years. For ceftazidime, resistance reached 64% in 2021 and increased to 94.6% in 2023, remaining above 90% in 2024 (*p* = 0.05). For cefepime, a similar and statistically significant upward trend was noted, with resistance rising from 64% in 2021 to 95% in 2024 (*p* < 0.05). These data indicate a rapid and sustained escalation of cephalosporin resistance among *A. baumannii* isolates within a three-year period. Trends in annual resistance to cephalosporins among the isolates are shown in Figure 2.

**Figure 3:**
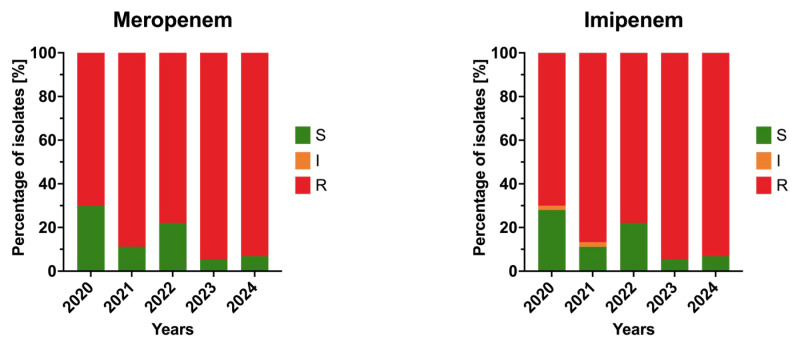
Annual resistance rates of *Acinetobacter baumannii* clinical isolates to carbapenems in 2020–2024. Bars represent proportions of susceptible (S), susceptible increased exposure (I), and resistant (R) isolates.

Carbapenems showed persistently high resistance throughout the study period. Meropenem resistance increased from approximately 70% in 2020 to 94.6% in 2023, while imipenem resistance rose from 70% to 93% over the same period, though these changes were not statistically significant (*p* > 0.05). Overall, *A. baumannii* isolates demonstrated nearly universal resistance to β-lactams by 2024. Trends in annual resistance to carbapenems among the isolates are presented in Figure 3.

### 3.3. Aminoglycosides

**Figure 4:**
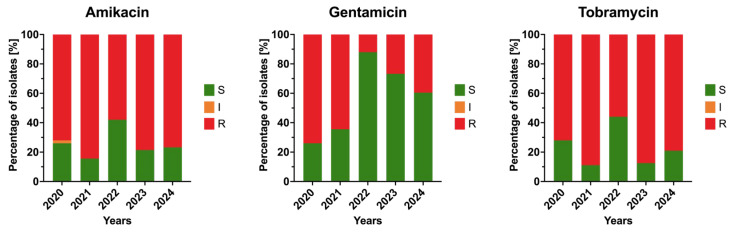
Annual resistance rates of *Acinetobacter baumannii* clinical isolates to aminoglycosides in 2020–2024. Bars represent proportions of susceptible (S), susceptible increased exposure (I), and resistant (R) isolates.

Resistance to amikacin remained high, fluctuating between 58% and 84%, without a significant trend over time (*p* > 0.05). Tobramycin showed a similar unstable pattern, with resistance varying between 56% and 88%. In contrast, gentamicin displayed a temporary improvement, with resistance decreasing from 74% in 2020 to 12% in 2022, followed by a moderate increase to 39% in 2024. Although this downward trend was not statistically significant, the data suggest a partial recovery of susceptibility to gentamicin in recent years. Trends in annual resistance to aminoglycosides among the isolates are shown in Figure 4.

### 3.4. Fluoroquinolones

**Figure 5:**
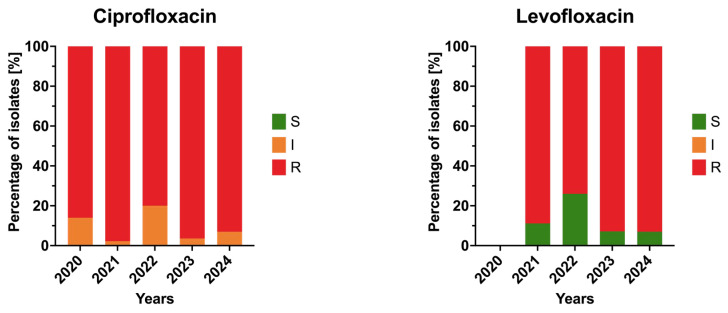
Annual resistance rates of *Acinetobacter baumannii* clinical isolates to fluoroquinolones in 2020–2024. Bars represent proportions of susceptible (S), susceptible increased exposure (I), and resistant (R) isolates.

Both ciprofloxacin and levofloxacin exhibited consistently very high resistance rates, exceeding 85% in all study years. Ciprofloxacin resistance fluctuated between 80% and 98%, while levofloxacin resistance reached up to 93% by 2024. No statistically significant trends were observed, indicating stable, persistently high resistance within this antibiotic class. The annual resistance profiles of the isolates to fluoroquinolones are presented in Figure 5.

### 3.5. Antifolates

**Figure 6:**
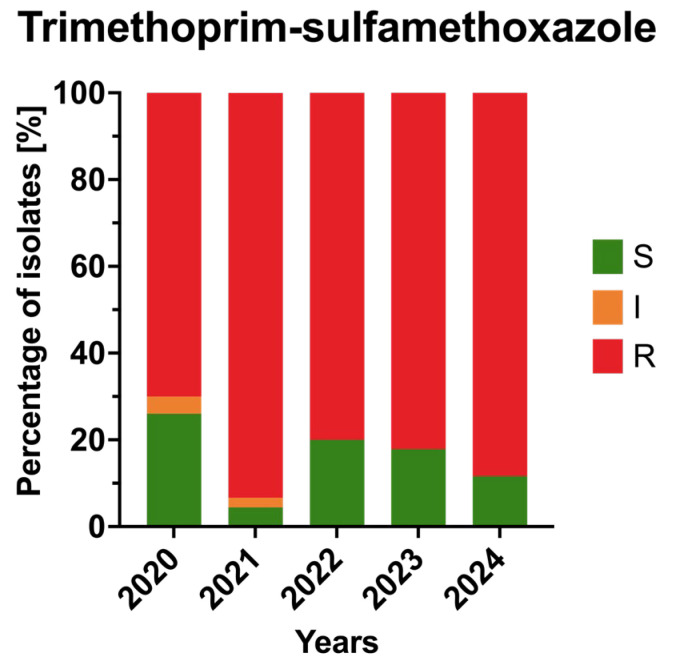
Annual resistance rates of *Acinetobacter baumannii* clinical isolates to antifolates in 2020–2024. Bars represent proportions of susceptible (S), susceptible increased exposure (I), and resistant (R) isolates.

Resistance to trimethoprim–sulfamethoxazole remained high throughout the study, ranging from 70% in 2020 to 88% in 2024. The trend showed minor year-to-year variability but no significant overall change (*p* > 0.05). This persistent resistance suggests limited clinical usefulness of trimethoprim–sulfamethoxazole against *A. baumannii* isolates in this setting. The annual resistance profiles of the isolates to trimethoprim–sulfamethoxazole are presented in Figure 6.

### 3.6. Glycylcyclines

**Figure 7:**
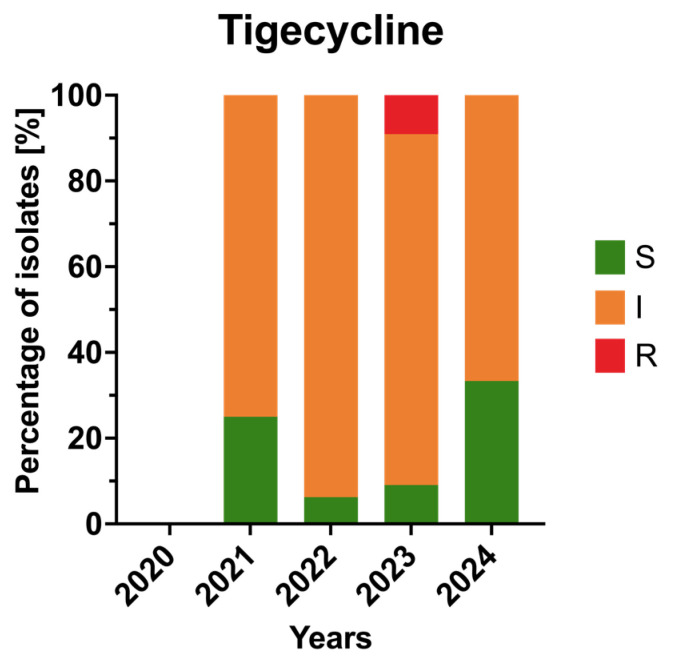
Annual resistance rates of *Acinetobacter baumannii* clinical isolates to glycylcyclines in 2020–2024. Bars represent proportions of susceptible (S), susceptible increased exposure (I), and resistant (R) isolates.

Tigecycline retained good activity against *A. baumannii*. Resistance was not detected in 2020–2022, appeared transiently in 2023 (9.1%), and again dropped to 0% in 2024. No significant trend was observed (*p* > 0.05). The annual resistance profiles of the isolates to tigecycline are presented in Figure 7.

### 3.7. Colistin

Over the five-year period, the proportion of CRAB isolates resistant to colistin remained low overall, with considerable year-to-year variation (Table 1). In 2020 and 2022, no colistin-resistant isolates were detected among CRAB (0% in both years; 95% confidence intervals [CI] up to ~10% in 2020 and ~9% in 2022). A notable transient increase in colistin resistance was observed in 2021, when 7 out of 40 CRAB isolates were colistin-resistant, corresponding to 17.5% (95% CI 7.3–32.8%). This peak was followed by a drop in 2022 (0/39 isolates). In 2023, only 1 colistin-resistant CRAB isolate was identified out of 53 (1.9%; 95% CI 0.0–10.1%). By 2024, the proportion of colistin-resistant CRAB rose again modestly to 7.5% (3/40 isolates; 95% CI 1.6–20.4%). Despite these fluctuations, including the 2021 spike, no consistent upward or downward trend in colistin resistance was evident over time. A Cochran–Armitage test for trend did not demonstrate a significant linear change in resistance rates across 2020–2024 (*p* > 0.7). Similarly, logistic regression treating year as a continuous variable showed no significant annual change in the odds of colistin resistance (odds ratio per year = 0.94, 95% CI 0.61–1.47, *p* = 0.79). In other words, the yearly variations appeared to be irregular rather than indicative of a sustained trend.

### 3.8. Mechanisms of Carbapenem Resistance

**Figure 8:**
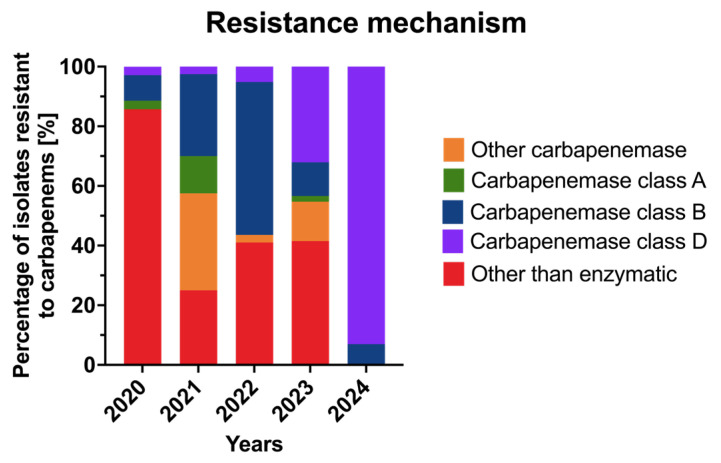
Distribution of carbapenem resistance mechanisms among *Acinetobacter baumannii* isolates in 2020–2024.

The distribution of carbapenem resistance mechanisms among *A. baumannii* isolates varied markedly throughout the study period (Figure 8).

In 2020, the dominant mechanism was non-enzymatic resistance, detected in 85.7% of isolates. Carbapenemases of class A and class D were each identified in 2.9%, while class B carbapenemases were present in 8.6% of isolates. No isolates producing unclassified carbapenemases were detected in that year. In 2021, a marked shift in the resistance pattern was observed. Non-enzymatic mechanisms accounted for 25% of isolates, while unclassified carbapenemases emerged in 32.5%. The prevalence of carbapenemase types was class A—12.5%, class B—27.5%, and class D—2.5%. In 2022, the class B carbapenemases became the most frequent mechanism, detected in 51.3% of isolates. Non-enzymatic resistance remained common (41.0%), while class D carbapenemases occurred in 5.1% and unclassified carbapenemases in 2.6% of isolates. Class A carbapenemases were not detected in that year. In 2023, the distribution shifted again, with non-enzymatic mechanisms persisting at 41.5%. The proportion of isolates producing class D carbapenemases increased to 32.1%, whereas unclassified carbapenemases, class B, and class A enzymes were identified in 13.2%, 11.3%, and 1.9% of isolates, respectively. In 2024, the resistance profile changed dramatically. Most isolates (93.0%) produced class D carbapenemases, while class B carbapenemases were detected in only 7.0%. No isolates exhibited solely non-enzymatic resistance mechanisms in that year. Overall, these data indicate a progressive replacement of non-enzymatic mechanisms by enzymatic ones, with a clear dominance of OXA-type (class D) carbapenemases by 2024. This evolution suggests clonal expansion or dissemination of OXA-producing *A. baumannii* strains within the hospital environment, consistent with trends observed in many European healthcare settings.

## 4. Discussion

*A. baumannii* is currently regarded as one of the most serious nosocomial pathogens worldwide, characterized by a high epidemic potential and a remarkable ability to acquire resistance to multiple classes of antibiotics [47,48]. This bacterium poses a particular challenge in intensive care units, where it contributes to increased mortality, prolonged hospitalization, and substantial healthcare costs [49,50]. In recent years, an alarming rise has been observed in the prevalence of multidrug-resistant (MDR), extensively drug-resistant (XDR), and pandrug-resistant (PDR) strains, which has significantly limited available therapeutic options [51,52].

Over the five-year observation period (2020–2024) at the Central Teaching Hospital of the Medical University of Łódź, a total of 244 clinical isolates of *A. baumannii* were identified. The annual number of isolates remained relatively stable, with minor fluctuations ranging from 43 to 56 isolates per year. The highest number of isolates was recorded in 2023 (56 isolates), and the lowest in 2024 (43 isolates). Similar epidemiological patterns have been reported in other European centers, where *A. baumannii* continues to be a significant clinical challenge, particularly in ICUs [49,53].

A study conducted in Greece between 2016 and 2023 found *A. baumannii* to be the second most frequently isolated pathogen among carbapenem-resistant Gram-negative bacteria, accounting for 19.73% of all ESKAPE isolates [50]. Likewise, research from Slovakia between 2019 and 2022 demonstrated that *A. baumannii* was among the most common ICU pathogens, with an increasing tendency toward XDR phenotypes [49].

It is noteworthy that, during the analyzed period, no marked increase in the number of *A. baumannii* isolates was observed in the Łódź hospital, which may indicate the effectiveness of implemented infection control measures. This observation is significant considering that numerous centers worldwide have reported an increase in the prevalence of this pathogen, particularly during the COVID-19 pandemic [54,55]. For instance, a study from Iran revealed a substantial increase in carbapenem-resistant *A. baumannii* isolates in ICUs during the three pandemic waves of COVID-19 [54,55].

During the 2020–2024 observation period at the Central Teaching Hospital of the Medical University of Łódź, *A. baumannii* isolates exhibited extremely low susceptibility to third- and fourth-generation cephalosporins, consistent with the well-established profile of this pathogen as being highly resistant to β-lactams. Literature reports have consistently documented a high proportion of *A. baumannii* strains resistant to ceftazidime and cefepime, often exceeding 90%. Similarly, in our regional data, most isolates were non-susceptible to these antibiotics, indicating that these cephalosporins offer virtually no therapeutic value in the treatment of *A. baumannii* infections. For example, in studies from Iran, resistance to ceftazidime and cefepime exceeded 90%, closely mirroring rates reported in India [56]. Slightly lower, yet still very high, resistance rates of 50–70% have been reported in some Asian studies [57], meaning that more than half of isolates remained non-susceptible. In countries such as Lebanon, cefepime efficacy has been alarmingly low, with susceptibility rates of only ~13% in 2011–2013 [58]. These data confirm that *A. baumannii* resistance to cephalosporins is a widespread global phenomenon.

The underlying causes of such high resistance rates include both intrinsic and acquired mechanisms. *A. baumannii* naturally produces AmpC-type cephalosporinases and class D OXA-51 β-lactamases, which together confer resistance to penicillins and most cephalosporins. Even wild-type strains are inherently resistant to cefazolin, cefotaxime, and ceftriaxone. This resistance is further enhanced by the presence of narrow-spectrum PSE-1 (*Pseudomonas* Specific Enzyme 1) penicillinases or ESBLs such as PER-1 and VEB-1, which can hydrolyze broad-spectrum cephalosporins. For instance, PER-1, known for its potent hydrolysis of ceftazidime, was first reported in *A. baumannii* strains from Turkey and significantly reduced the clinical utility of third-generation cephalosporins. Additionally, *A. baumannii* is characterized by low outer membrane permeability and active drug efflux, further reducing cephalosporin efficacy [59]. Collectively, these factors mean that most hospital *A. baumannii* strains are intrinsically multidrug-resistant, and cephalosporins, including ceftazidime and cefepime, are ineffective in therapy.

Clinically, this implies that such antibiotics should not be used empirically in severe infections suspected to be caused by *A. baumannii*. Our local findings support this position: the high prevalence of MDR and XDR strains excludes cephalosporins as first-line agents. The situation is analogous in other regions; e.g., in Lebanon cefepime efficacy has dropped below 20%, while in Asia and the Middle East the proportion of MDR *A. baumannii* strains reaches 90% [58]. Regional differences may stem from varying antibiotic consumption patterns; countries with high cephalosporin and carbapenem use have applied strong selective pressure, leading to the emergence of clones virtually untreatable with these drugs [59]. Therefore, strict monitoring of susceptibility patterns and avoiding cephalosporin overuse are essential to preventing further resistance escalation.

From a clinical standpoint, the high resistance rates necessitate a revision of empirical treatment protocols for HAIs. *A. baumannii* frequently requires alternative therapy. Historically, carbapenems served as last-resort agents, but growing resistance to them has also narrowed treatment options. In targeted therapy, cephalosporins rarely have a role against *A. baumannii*, with potential exceptions being novel β-lactam/β-lactamase inhibitor combinations or cefiderocol. It remains important for microbiology laboratories to continue testing cephalosporin susceptibility, as such results contribute to defining MDR profiles and detecting possible shifts in resistance trends, whether due to new mechanisms or reduced resistance following antibiotic stewardship interventions. However, in the current reality of our hospital and many others worldwide, ceftazidime and cefepime are no longer effective options and serve primarily as indicators of the magnitude of the *A. baumannii* growing antimicrobial resistance problem.

For decades, carbapenems have formed the cornerstone of therapy for severe infections caused by *A. baumannii*. However, since the mid-2010s, a dramatic global increase in resistance to this class of drugs has been observed [59,60]. Our local data from the hospital indicate a very high proportion of isolates resistant to imipenem and meropenem, particularly during the COVID-19 pandemic. This finding is consistent with national and European trends. In Poland, between 2017 and 2019, over 70% of *A. baumannii* isolates were carbapenem-resistant, compared to rates of up to 38% in 2012. In our hospital, a similar upward trend was noted, while rates were likely somewhat lower in the pre-pandemic period, a sharp increase occurred during the pandemic (2020–2021), with CRAB strains accounting for over 80% of all isolates. This pattern was also reported in other centers in Poland; for example in Wrocław the proportion of carbapenem-resistant *A. baumannii* rose from 25–30% in 2019 to as high as 76–79% in 2021, a trend attributed to intensive antibiotic use during the pandemic and the greater number of severely ill patients [59]. European EARS-Net statistics confirm this association: countries with baseline CRAB prevalence ≥50% experienced a significant increase in *A. baumannii* bacteremia (+57%) in 2020–2021 compared to 2018–2019. Most infections originated in ICUs, underscoring the role of *A. baumannii* as an opportunistic pathogen in settings of intensive care and invasive procedures [61].

Geographically, carbapenem resistance in *A. baumannii* varies by region but remains a major concern everywhere. The average CRAB rate across Europe in 2013–2017 was 35.6%, but the distribution was highly uneven. In southern and eastern Europe, including the Mediterranean basin, the Balkans, and Central and Eastern Europe (including Poland) rates reached 72–76%, whereas in northern and western Europe, including Scandinavia and Germany, rates remained in the single digits [60]. Poland is unfortunately among the high-endemicity countries for CRAB, with pre-pandemic values already exceeding 50%, and local reports indicate further increases. Outside Europe, the situation is equally concerning in Asia (China, India) and the Middle East, where many centers report >80–90% resistance to imipenem or meropenem. In Indian studies, over 90% of isolates were carbapenem-resistant, with similar figures in Iran [56]. In Turkey and Mediterranean countries, the late 2010s saw near-complete dominance of XDR strains, susceptible only to colistin [62]. In contrast, some regions with historically low *A. baumannii* prevalence, mainly Scandinavia, still face only sporadic cases, though there remains a risk of importing resistant clones from endemic areas [60].

The predominant mechanism of carbapenem resistance in *A. baumannii* is the production of carbapenemases. These may belong to Ambler class D, class B, or, less commonly, class A [59]. The most clinically and epidemiologically significant are class D OXA-type β-lactamases, particularly OXA-23, OXA-24/40, and OXA-58, which are globally widespread and account for most carbapenem resistance cases in this species [62]. OXA-51 is a chromosomally encoded, intrinsic carbapenemase in *A. baumannii*, but alone it typically confers only low-level resistance, and clinically relevant resistance arises from overexpression due to an upstream ISAba1 promoter or co-production with additional enzymes [62]. OXA-23, OXA-24/40, and OXA-58 are the most frequently detected carbapenemases in CRAB isolates worldwide and are responsible for most outbreaks in Europe and Asia. In Poland, hospital isolates have also been shown to carry *bla_OXA_* genes; e.g., in studies performed from 2009 *bla_OXA-23_* was predominant [63]. Class B carbapenemases, such as NDM-1, VIM, and IMP, are less frequent in *A. baumannii* than in *Enterobacterales*, but their importance is increasing. In China, approximately 60% of *A. baumannii* strains are now carbapenem-resistant, partly due to the spread of *bla_NDM-1_*. In Europe, NDM-producing CRAB outbreaks have been reported, particularly in Balkan countries. Class A carbapenemases, especially KPC, are rare in *A. baumannii*, though they have been described in Greece, Italy, and the USA [64]. An interesting example involves GES-type enzymes detected in Turkish isolates alongside OXA-23; for example GES-11 and GES-22 co-occurred with OXA, thus further enhancing resistance [62]. This demonstrates *A. baumannii’s* ability to accumulate multiple resistance mechanisms simultaneously.

Carbapenem resistance is often multifactorial. In addition to carbapenemase production, *A. baumannii* can employ non-enzymatic mechanisms that potentiate resistance, meaning that even moderately active OXA-23 production, combined with outer membrane changes, can yield a full resistance phenotype [59]. Polish studies confirm that only a proportion of CRAB isolates carry acquired carbapenemases; the remainder, despite lacking detectable MBL or KPC enzymes, are resistant due to membrane-associated mechanisms. For example, in Wrocław (2021–2022), around 20–25% of carbapenem-resistant *A. baumannii* isolates did not produce MBLs, indicating the contribution of non-enzymatic mechanisms [59]. The combined presence of enzymatic and non-enzymatic mechanisms is particularly concerning, as it leads to the emergence of XDR and even PDR strains.

The clinical implications of carbapenem resistance are severe. Drugs once considered last-resort agents (imipenem, meropenem) are now frequently ineffective. In settings where over 80–90% of hospital isolates are resistant, carbapenems cannot be used empirically for suspected *A. baumannii* infections. Reports indicate that CRAB infections are associated with significantly higher mortality compared to infections caused by susceptible strains [57]. In one study, mortality in CRAB infections reached 63%, with most deaths attributable to sepsis [58]. Given such poor outcomes, the prompt initiation of effective targeted therapy is crucial, and combination therapy is increasingly recommended for *A. baumannii* infections to avoid monotherapy failures. Evidence suggests that combining two active agents may improve survival and slow the emergence of further resistance [65].

In summary, the local and global rise in carbapenem resistance represents a critical epidemiological challenge. Addressing it requires intensified infection control efforts, CRAB carrier isolation, rigorous hospital hygiene, rational antibiotic use, and the development of new therapies. Data from our hospital reflect this broader pattern; the pandemic likely exacerbated the problem, but even after its peak, endemic resistance levels remain high, necessitating ongoing surveillance and regular updates to local therapeutic guidelines.

Aminoglycosides have historically shown some activity against *A. baumannii*; however, an upward trend in resistance has been observed. In our study, *A. baumannii* isolates exhibited high levels of resistance to gentamicin, amikacin, and tobramycin. Similar observations were made by a team in Wrocław, where the proportion of isolates resistant to amikacin increased from 34% in 2019 to 71% in 2021, and gentamicin resistance rose from 22% to 77% during the same period. This means that, at the height of the pandemic, nearly three out of four *A. baumannii* isolates were non-susceptible to aminoglycosides. National data indicate that resistance to these drugs has remained at around 73% in recent years [59]. Globally, reports also document very high rates of aminoglycoside resistance; e.g., a meta-analysis covering the period 1995–2023 found an average resistance rate of approximately 70–80% [66]. In some countries, the situation is even more severe; for instance in India over 90% of isolates were resistant to gentamicin and amikacin. In certain Asian populations, resistance to amikacin was somewhat lower in 2010, at 50–60% in China, but the overall trend has been upward [56].

Resistance mechanisms in *A. baumannii* to aminoglycosides are complex and multifactorial. First, the bacterium can acquire aminoglycoside-modifying enzymes that inactivate the drug through adenylation, acetylation, or phosphorylation. Clinical isolates frequently carry genes encoding such enzymes, e.g., Turkish isolates possessed the *aac(3)-Ia* and *aac(6′)-Ib* acetyltransferase genes in 13% and 15% of isolates, respectively [62]. The presence of these enzymes correlates with resistance to gentamicin and tobramycin. In addition, *A. baumannii* can harbor 16S rRNA methyltransferases, such as ArmA and RmtD, which methylate the aminoglycoside binding site on the ribosome, conferring high-level resistance to all aminoglycosides. Methyltransferase genes have been detected in European *A. baumannii* clones since around 2010, explaining the sudden emergence of isolates completely resistant to amikacin despite the absence of typical modifying enzymes. Another factor is the activity of RND efflux systems that expel aminoglycosides from the cell, reducing their intracellular concentration. While the primary AdeABC efflux system in *A. baumannii* has a greater impact on drugs such as tigecycline and fluoroquinolones, its overactivity can also moderately increase aminoglycoside MIC values [57].

When comparing our local situation to other regions, certain differences are apparent, possibly reflecting varying treatment practices. In many countries with very high carbapenem resistance rates such as in the Mediterranean and Middle East, aminoglycosides have continued to be used in combination therapy, which may have paradoxically helped maintain slightly lower resistance rates than those observed for β-lactams. Nevertheless, even in these regions, resistance levels are now converging; in Lebanon, approximately 80–90% of isolates are resistant to amikacin. In one Lebanese study, all isolates remained susceptible to colistin and tigecycline, underscoring that aminoglycosides are no longer effective and that the active agents of choice have shifted to polymyxins and glycylcyclines [58]. In Poland, aminoglycosides have long been disfavored for treating *A. baumannii* infections, used mainly as adjunctive therapy or when no other options were available. Limited use may have slowed the development of resistance, explaining why, until the late 2010s, around 50% of isolates remained susceptible to amikacin [59]. During the pandemic, intensified antibiotic use, including frequent empirical aminoglycoside administration in critically ill patients, may have accelerated the selection of resistant strains, reflected in the 2021 resistance increase to 70% [59].

Clinically, rising *A. baumannii* resistance to aminoglycosides limits the utility of this drug class. Just over a decade ago, amikacin was considered one of the more active agents against *A. baumannii* and was recommended as part of combination regimens for severe pneumonia. Today, however, with three-quarters of isolates resistant, the effectiveness of such an approach is doubtful. Empirical aminoglycoside therapy (e.g., with gentamicin) is not advisable for suspected *A. baumannii* infections under these resistance conditions. In practice, aminoglycosides should be considered only for targeted therapy, if the isolate is confirmed susceptible, and when pharmacokinetic advantages exist (e.g., urinary tract infections where high local concentrations are achieved). In severe bloodstream or respiratory infections, aminoglycosides play only a limited role, primarily as part of combination therapy for potential synergistic effects with β-lactams or polymyxins. Given the ongoing rise in resistance, monitoring the prevalence of genes such as *armA* in hospital populations is essential, as their emergence signals the practical loss of the entire aminoglycoside class as a treatment option [67]. In conclusion, both local and global data suggest that aminoglycosides should no longer be considered first-line agents against *A. baumannii*, with use restricted to cases where susceptibility is confirmed, and benefits outweigh toxicity risks.

Although fluoroquinolones remain active against many Gram-negative bacteria, they unfortunately show the lowest efficacy against *A. baumannii* of all conventional antibiotic classes. In our local observations, virtually all *A. baumannii* isolates were resistant to ciprofloxacin and levofloxacin in 2020–2024. Even prior to the pandemic, susceptibility to these drugs was very low, and the situation worsened during the pandemic alongside increased empirical fluoroquinolone use. This trend is mirrored by data from other Polish centers. In Wrocław, *A. baumannii* ciprofloxacin resistance rose from 48% in 2019 to 87% in 2021 and reached 100% in 2022 [59]. Levofloxacin resistance followed a similar pattern; after a transient decrease to 45–53% in 2019–2020, it rose in 2021, and by 2022, 63% of isolates were resistant. Nationally, fluoroquinolone resistance in *A. baumannii* has reached 87% in recent years, one of the highest rates among all antibiotic classes [59]. Globally, the situation is equally concerning, as reviews report consistently high rates of ciprofloxacin resistance, frequently exceeding 70% across multiple regions. In some studies, resistance rates exceeded 90%, highlighting the severity of this problem [51,68,69]. In the Middle East, the situation is similarly severe in Lebanon ciprofloxacin and levofloxacin were virtually ineffective, with one study finding that all MDR *A. baumannii* isolates were resistant to these agents [58]. Global averages suggest that over 70% of *A. baumannii* strains are non-susceptible to fluoroquinolones, rendering this class almost useless for treating infections caused by this pathogen [70].

The high and rising resistance of *A. baumannii* to fluoroquinolones stems from well-characterized genetic and selective mechanisms. First, the organism readily acquires mutations in the *gyrA* and *parC* genes encoding DNA gyrase and topoisomerase IV, the target enzymes of fluoroquinolones. Point mutations, such as a serine-to-leucine substitution at positions analogous to *E. coli gyrA*, markedly increase ciprofloxacin MIC values. Most ciprofloxacin-resistant clinical isolates harbor at least one such mutation, and studies have shown that QRDR (quinolone resistance-determining region) mutations are widespread in epidemic *A. baumannii* clones [71]. Second, activation of RND efflux systems, particularly the AdeABC pump, reduces intracellular fluoroquinolone concentrations. While AdeABC overexpression is primarily associated with tigecycline resistance, it also contributes to ciprofloxacin resistance [57]. Strains with hyperactive AdeABC pumps often display multidrug resistance including fluoroquinolones, correlating with the XDR phenotype. Third, *A. baumannii* has inherently low susceptibility to certain fluoroquinolones (e.g., norfloxacin) due to low outer membrane permeability and the presence of the AdeIJK pump, another RND efflux system with broad substrate specificity.

A critical factor accelerating the selection of fluoroquinolone-resistant strains is the overuse of these drugs. Early in the COVID-19 pandemic, fluoroquinolones, especially levofloxacin, were widely administered empirically to patients with suspected bacterial co-infection, increasing selective pressure on hospital flora. In Wrocław, increased fluoroquinolone consumption in 2020 correlated with subsequent *A. baumannii* resistance growth. Even after stewardship programs reduced fluoroquinolone use, no substantial improvement in susceptibility was observed, indicating that once resistant clones are established, they become entrenched in the hospital population. This underscores an important lesson: prudent antibiotic use must prevent the widespread emergence of resistant strains, as reversing such trends is extremely difficult [59].

From a clinical perspective, fluoroquinolones currently play no significant role in *A. baumannii* therapy. Susceptibility rates are so low that these drugs are not recommended for either empirical or targeted treatment, except in rare cases where antibiograms confirm full susceptibility, usually in a few environmental isolates. Moreover, fluoroquinolones can promote cross-resistance; for example ciprofloxacin use may select not only for fluoroquinolone-resistant strains but also for carbapenem-resistant ones, due to the frequent co-occurrence of *gyrA* mutations with *bla_OXA_* genes on mobile genetic elements. Exposure to fluoroquinolones may therefore promote the persistence of such resistance determinants [63].

Trimethoprim–sulfamethoxazole (TRS) is a broad-spectrum antimicrobial occasionally used for bacterial infections, including in rare cases against *A. baumannii* strains retaining susceptibility. However, as with other drug classes, susceptibility rates have steadily declined. In this study, *A. baumannii* isolates exhibited high resistance to antifolate agents during 2020–2024, consistent with global trends. In European and Asian countries over the past decade, TRS resistance in *A. baumannii* has frequently exceeded 70% [57]. For example, in the study by Chen et al., 71.3% of isolates were resistant [70]. Resistance rates in Iran reached 85%, in India 73–80%, and in China approximately 61% [56]. Poland does not differ from these statistics. *A. baumannii* displays high intrinsic tolerance to trimethoprim and sulfonamides, meaning that even in the absence of acquired resistance genes, isolates exhibit considerable non-susceptibility. According to literature data, susceptibility in Poland has long been limited; as early as 2012–2013, fewer than 30% of isolates remained susceptible, and the situation has likely worsened since [59].

Resistance to trimethoprim and sulfonamides in *A. baumannii* arises from alternative folate synthesis pathways or efficient folate uptake from the environment, rendering TRS inhibition less effective [59]. In addition, *A. baumannii* frequently harbors mobile genetic elements carrying sulfonamide resistance genes (*sul1*, *sul2*) and trimethoprim resistance genes (*dfrA*) encoding dihydrofolate reductases insensitive to trimethoprim. The *sul1* and *sul2* genes are often embedded in class 1 integrons found in MDR *A. baumannii* worldwide. Their presence confers high sulfamethoxazole MICs and renders the drug clinically ineffective. *dfr* genes are also occasionally detected in integrons, albeit less frequently than *sul* genes. These integrons often co-carry aminoglycoside resistance genes, facilitating co-selection. As a result, strains resistant to carbapenems and fluoroquinolones are almost always resistant to TRS as well, contributing to the MDR or XDR phenotype [1]. Efflux mechanisms may also play a minor role in reducing intracellular trimethoprim concentrations, although this is not the primary resistance pathway.

From an epidemiological perspective, resistance of A. baumannii to trimethoprim–sulfamethoxazole shows notable geographical variation. In certain Asian countries, moderate susceptibility rates, reaching up to approximately 40% of isolates, have been reported in the past decade. [51,69]. This variability has been attributed, among other factors, to differences in antimicrobial usage patterns and the distribution of clones lacking sul1-associated resistance determinants. In contrast, in the Indian subcontinent and the Middle East, high resistance levels (70–80%) have been associated with widespread use of trimethoprim–sulfamethoxazole in outpatient settings, particularly for skin and urinary tract infections, which may contribute to increased selective pressure. In Western Europe, despite more limited use of this agent, resistance rates remain high, likely reflecting the circulation of resistant clones originating from endemic regions, with reported rates exceeding 70% in countries such as Spain and Italy [56].

In clinical practice, trimethoprim–sulfamethoxazole plays only a limited role in the management of *A. baumannii* infections. Isolated reports describe its use in skin and soft tissue infections or urinary tract infections caused by multidrug-resistant (MDR) *A. baumannii*, particularly when no other oral treatment options are available [72]. However, given the high prevalence of resistance, empirical use of trimethoprim–sulfamethoxazole carries a substantial risk of therapeutic failure. At the Central Teaching Hospital of the Medical University of Łódź, local resistance data suggest that trimethoprim–sulfamethoxazole is unlikely to be included in standard treatment regimens for *A. baumannii* infections. Exceptions may occur in rare cases of susceptible isolates, for example in localized urinary tract infections in clinically stable patients, where oral therapy would be advantageous. Overall, trimethoprim–sulfamethoxazole belongs to a group of agents with marginal efficacy against *A. baumannii*, and its clinical utility continues to decline in parallel with the progression of antimicrobial resistance. Unfortunately, the global trend indicates that *A. baumannii* has virtually lost susceptibility to trimethoprim–sulfamethoxazole through the acquisition of a broad array of resistance genes, a pattern confirmed by both our local observations and the literature from the past decade.

Tigecycline, a glycylcycline-class antibiotic, has been one of the few effective therapeutic options against *A. baumannii* strains resistant to other major antibiotic groups for over a decade. Its mechanism of action, inhibition of protein synthesis via binding to the 30S ribosomal subunit, circumvents many common resistance pathways, and initially the vast majority of *A. baumannii* strains were susceptible. Indeed, in the early years following its introduction (2005–2010), in vitro susceptibility exceeded 90%. Unfortunately, with broader clinical use, isolates exhibiting elevated resistance began to emerge. Our local data from 2020–2024 indicate that tigecycline remained relatively effective against *A. baumannii*, although a downward trend in susceptibility was evident. The majority of MDR isolates at our hospital were still susceptible: approximately 70–80% which aligns with literature data. In a previously cited multicentre study, resistance to tigecycline was observed in 8.5% of isolates, while 26.7% were classified as intermediate, leaving approximately 65% of isolates susceptible [70]. Similar patterns have been reported in many countries across Europe and Asia, where susceptibility rates range from 60–80%. For example, in Spain and Italy between 2014 and 2017, around 70% of *A. baumannii* isolates remained susceptible to tigecycline despite high carbapenem resistance rates [73]. Even higher rates were observed in parts of the Middle East; in Lebanon in 2021 all isolates from one hospital were tigecycline-susceptible [58], likely reflecting careful restriction of the drug as a last-resort therapy. Conversely, in countries with heavy tigecycline use, resistance rates have been rising; in certain centres in Greece and Turkey, the proportion of resistant strains reached 30–40% even before 2020. In Poland, most *A. baumannii* isolates remain tigecycline-susceptible, although intermediate (susceptible, increased exposure) and resistant phenotypes are occasionally detected. These often overlap with colistin-resistant isolates, suggesting an extreme XDR or PDR profile [74].

Resistance mechanisms to tigecycline in *A. baumannii* are distinctive, with a central role played by the overexpression of resistance–nodulation–division (RND) efflux pumps, particularly AdeABC. Clinical isolates with elevated tigecycline MICs often carry mutations or deletions in the adeN gene, which encodes a repressor regulating AdeABC expression [75,76]. Such mutations result in constitutive overproduction of the pump and active extrusion of tigecycline from the cell. Hornsey et al. reported a direct correlation between high tigecycline MICs (resistance) and increased *adeB* expression, the gene encoding a key structural component of the AdeABC system [76]. Functionally active AdeABC pumps can export tigecycline before it reaches and inhibits the ribosome. Importantly, this resistance mechanism often arises following exposure to the drug, with in vivo selection documented in patients receiving prolonged tigecycline therapy. Consequently, treatment guidelines recommend using tigecycline in combination with ampicillin-sulbactam to reduce the risk of selecting resistant mutants [2].

Clinically, tigecycline remains a valuable option for treating *A. baumannii* infections, particularly MDR and XDR phenotypes. Its broad spectrum covers non-fermenting Gram-negative bacilli, and it achieves good tissue penetration, making it useful for severe infections such as skin and soft tissue infections. However, tigecycline has limitations: it is bacteriostatic and achieves relatively low serum concentrations, making it unsuitable for bloodstream infections or central nervous system infections caused by *A. baumannii*. Our local findings of sustained tigecycline activity support its continued use, provided strict monitoring is in place. Clinicians should closely observe treatment response, as the emergence of tigecycline resistance during therapy typically presents as clinical deterioration or lack of improvement, warranting escalation of treatment.

In summary, among the antibiotic classes reviewed, tigecycline remains one of the more active agents against *A. baumannii*. Both local and global data show that most isolates remain susceptible, though the downward trend is clear [70]. The emergence of fully tigecycline-resistant strains would be a highly alarming development, signaling near-complete PDR. For this reason, ongoing surveillance and judicious, de-escalation-based use of tigecycline are essential to preserving its efficacy as a “last-resort” treatment in the face of growing resistance to almost all other available antibiotic classes.

Polymyxins, particularly colistin (polymyxin E), have represented the last line of defense against *A. baumannii* infections for over a decade. Colistin acts by disrupting the structure of lipopolysaccharide (LPS) in the bacterial outer membrane, leading to membrane damage and cell death. For many years, colistin resistance in *A. baumannii* was considered rare, and even a decade ago, most XDR strains remained susceptible [1]. Unfortunately, the increased use of sodium colistimethate for MDR infections has led to a “renaissance” of this drug but also created selective pressure favouring resistant isolates. In our local data from 2020–2024, *A. baumannii* isolates were generally colistin-susceptible, although some variation in MIC values was observed. Analysis of colistin MIC distribution suggests a possible upward drift MIC creep, an early signal of selection for less susceptible subpopulations. EUCAST clinical breakpoints for colistin against *Acinetobacter* are MIC ≤ 2 mg/L (susceptible) and >2 mg/L (resistant) [77]. Literature reports indicate that many isolates now present MICs at 2 mg/L, whereas earlier, values ≤ 0.5–1 mg/L were predominant [78]. In our setting, a few isolates with elevated MICs formally classified as resistant were also detected. The relatively high proportion of colistin-resistant isolates observed in 2021 (17.5%) should be interpreted with caution due to the limited sample size and wide confidence interval. This finding may reflect a transient local cluster or fluctuation rather than a sustained epidemiological trend, as resistance rates returned to low levels in subsequent years.

Globally, the prevalence of colistin resistance in *A. baumannii* remains relatively low compared to other antibiotic classes but shows a gradual upward trend. A meta-analysis of studies performed from 2010 to 2020 estimated the global mean resistance rate at approximately 5–10% [78]. However, certain regions, particularly Southeast Asia and countries in the eastern Mediterranean basin, report higher rates. According to Pormohammad et al. (2020), the average resistance rate to polymyxins in these areas was 11.2%, with the highest values observed in Lebanon (17.5%) and China (11.8%), and the lowest in Germany (0.2%) [79]. These figures underscore the marked geographical variability. In Europe, colistin retained its effectiveness for many years; reports from Germany and Scandinavia indicated virtually no resistance, with 0–1% of isolates resistant [79]. Unfortunately, southern European countries such as Greece and Italy began to report resistant cases, associated with the intensive use of polymyxins since the early 2000s. In Poland, national surveillance data place the country among those with low colistin resistance rates; recent reports from the National Antibiotic Protection Programme do not indicate a significant proportion of resistant *A. baumannii* isolates and most laboratories do not report such cases, or they are single, sporadic events [80]. This is consistent with a study from Wrocław covering 2017–2022, in which no *A. baumannii* isolates demonstrated phenotypic colistin resistance [59].

The mechanisms of colistin resistance in *A. baumannii* differ fundamentally from those for most other antibiotic classes, as no classic inactivating enzymes or efflux systems are involved. The primary routes are either modification or loss of the target binding site for colistin. Two major mechanisms are recognized: complete loss of lipopolysaccharide (LPS) and modification of lipid A, the lipid component of LPS. Some resistant strains carry mutations in the LPS biosynthesis pathway genes (*lpxA*, *lpxC*, *lpxD*), leading to a complete absence of LPS in the outer membrane [81]. LPS-deficient bacteria become colistin-resistant because the antibiotic no longer has a binding site; a classic example is *A. baumannii* with a *lpxD* deletion [82]. The second mechanism involves modification of lipid A through the addition of positively charged moieties, such as phosphoethanolamine or 4-amino-arabinose, which reduce the affinity of polymyxin E for LPS. These modifications are typically triggered by mutations in the regulatory genes *pmrA*/*pmrB*, which induce expression of enzymes adding these groups to lipid A. In *A. baumannii*, *pmrB* mutations have been linked to resistance despite the presence of structurally intact LPS [79,83]. Both mechanisms are chromosomally encoded and arise de novo under selective pressure. Importantly, unlike in *Enterobacterales*, *A. baumannii* has not demonstrated widespread dissemination of plasmid-mediated mcr genes capable of conferring transferable colistin resistance, and isolated reports of *mcr-4* detection in *A. baumannii* do not indicate an emerging trend [1]. This suggests that resistance in this species primarily arises through intrinsic mutations rather than horizontal gene transfer.

Biological trade-offs have been observed in colistin-resistant *A. baumannii*. Strains lacking LPS may exhibit reduced virulence and survival capability yet can still cause infections in critically ill patients. Moreover, such strains may show increased susceptibility to other antibiotics, such as carbapenems or tigecycline, due to altered membrane permeability [78]. Clinically, however, the emergence of colistin-resistant *A. baumannii* remains a major concern, often signaling a pathogen with a PDR (pan-drug-resistant) phenotype.

From a clinical standpoint, colistimethate sodium remains one of the last effective agents against *A. baumannii*, though its use poses significant challenges. The drug carries substantial nephrotoxicity and neurotoxicity, complicating safe dosing. Standard regimens require careful renal function monitoring and dose adjustments. High MIC values within the susceptible range (e.g., 2 mg/L) are also concerning, as they may necessitate higher doses, increasing toxicity risk. Consequently, invasive *A. baumannii* infections are often treated with polymyxins in combination with other agents, not only for potential synergistic effects, but also to allow lower colistin doses. Such combination therapy may delay resistance emergence, as the pathogen would need to simultaneously develop resistance to multiple drugs.

In summary, colistin remains an effective weapon against *A. baumannii* in our centre and in many regions worldwide, but its efficacy is under threat from resistance mechanisms involving LPS loss or modification and, potentially, plasmid-mediated mcr genes. Our local data confirm full or near-complete colistin activity, which is encouraging. Maintaining this status will require prudent use of colistimethate sodium and measures to prevent the introduction of resistant strains from external sources.

## 5. Conclusions

The five-year surveillance of *Acinetobacter baumannii* clinical isolates revealed a dynamic shift in resistance mechanisms, with a marked rise in OXA-type carbapenemase production replacing non-enzymatic pathways as the predominant mode of carbapenem resistance. Despite persistently high resistance rates to most antimicrobial classes, colistin susceptibility remained largely preserved among CRAB isolates. The observed temporal patterns underscore the evolutionary plasticity of *A. baumannii* in response to antimicrobial pressure and highlight the importance of continued local epidemiological monitoring to inform empirical treatment strategies and infection control policies in healthcare settings.

## Figures and Tables

**Table 1:** Colistin susceptibility of carbapenem-resistant *A. baumannii* (CRAB) by year (2020–2024). Resistant isolates are those with colistin MIC ≥ 2 mg/L (EUCAST criteria). 95% confidence intervals for the percentage resistant were calculated by exact binomial method.

Year	CRAB Isolates Tested (n)	Colistin-Resistant (n)	Percentage Resistant (95% CI)
2020	35	0	0.0% (0.0–10.0%)
2021	40	7	17.5% (7.3–32.8%)
2022	39	0	0.0% (0.0–9.0%)
2023	53	1	1.9% (0.0–10.1%)
2024	40	3	7.5% (1.6–20.4%)

## Data Availability

The dataset is available on request from the authors.

## Abbreviations

The following abbreviations are used in this manuscript:

ATCC	American Type Culture Collection
CDC	Centers for Disease Control and Prevention
CHCA	Alpha-cyano-4-hydroxycinnamic acid
CIM	Carbapenem Inactivation Method
CLSI	Clinical and Laboratory Standards Institute
CRAB	Carbapenem-Resistant *Acinetobacter baumannii*
ECDC	European Centre for Disease Prevention and Control
ESBL	Extended-Spectrum Beta-Lactamases
ESKAPE	*Enterococcus faecium*, *Staphylococcus aureus*, *Klebsiella pneumoniae*, *Acinetobacter baumannii*, *Pseudomonas aeruginosa*, *Enterobacter* spp.
EUCAST	European Committee on Antimicrobial Susceptibility Testing
FDA	Food and Drug Administration
GES	Guiana Extended-Spectrum β-Lactamase
IDSA	Infectious Diseases Society of America
IMP	Imipenemase
KORLD	National Reference Centre for Antimicrobial Susceptibility Testing
KPC	*Klebsiella pneumoniae* Carbapenemase
LPS	Lipopolysaccharide
MALDI-TOF MS	Matrix-Assisted Laser Desorption/Ionization Time-of-Flight Mass Spectrometry
MBL	Metallo-β-Lactamase
MDR	Multidrug-Resistant
MIC	Minimum Inhibitory Concentration
NDM	New Delhi Metallo-β-Lactamase
NSBL	Narrow-Spectrum Beta-Lactamases
OMV	Outer Membrane Vesicles
OXA	Oxacillinases
PBP	Penicillin-Binding Proteins
PER	Pseudomonas Extended-Resistance β-Lactamase
PDR	Pandrug-Resistant
PSE	Pseudomonas-Specific Enzyme
RND	Resistance-Nodulation-Division
SIM	Seoul Imipenemase
VAP	Ventilator-Associated Pneumonia
VEB	Vietnamese Extended-Spectrum β-Lactamase
VIM	Verona Integron-Encoded Metallo-β-Lactamase
WHO	World Health Organization
XDR	Extensively Drug-Resistant
